# Pseudoaneurysm Formation in a Pediatric Patient After Non-Traumatic Middle Cerebral Artery Dissection With a Rapid Spontaneous Complete Thrombosis

**DOI:** 10.7759/cureus.32251

**Published:** 2022-12-06

**Authors:** Orlando De Jesus, Fausto Lugo Morales, Juan C Vicenty

**Affiliations:** 1 Neurosurgery, University of Puerto Rico, Medical Sciences Campus, San Juan, PRI

**Keywords:** thrombosis, pseudoaneurysm, pediatric, middle cerebral artery, endovascular, dissecting, aneurysm

## Abstract

Spontaneous cerebral dissections in children are rare and can be associated with the formation of pseudoaneurysms. The management of these pseudoaneurysms is controversial as they can be treated either by surgery or endovascular techniques. On rare occasions, they may spontaneously thrombose. We present a 12-year-old male without a history of trauma who developed an intracerebral hematoma secondary to a ruptured pseudoaneurysm of the middle cerebral artery that showed a rapid spontaneous complete thrombosis. Five days after his initial diagnostic cerebral digital subtraction angiogram, a follow-up study showed no evidence of the previously observed pseudoaneurysm. Two months later, a computed tomographic angiography of the brain showed no evidence of the pseudoaneurysm. Thrombosed pseudoaneurysms should be closely followed by neuroimaging studies as they may subsequently recanalize.

## Introduction

Spontaneous cerebral dissections in children are rare [1]. These dissections can be associated with the formation of dissecting aneurysms or pseudoaneurysms, which may rupture and produce intracerebral hematomas. Pseudoaneurysms account for only 1% of all adult intracranial aneurysms [2]. However, the incidence is significantly higher in pediatric patients, estimated between 19%-24% of all pediatric intracranial aneurysms [3,4]. There is a male predominance in pediatric cases [3]. The most common risk factor in children is a history of head trauma [2,3]. The principal pathological findings of pseudoaneurysms involve the initial damage of the arterial wall internal elastic laminae progressing to the entire arterial wall, subsequently forming an organized encapsulated hematoma within a false cavity contained by fibrotic tissue in direct communication with the ruptured artery [2,5-7].

The management of these cerebral pseudoaneurysms is controversial as they can be treated either by surgery or endovascular techniques [3]. On rare occasions, they can be managed conservatively, as they can sometimes spontaneously thrombose [2,8,9]. We managed a 12-year-old male without a history of trauma who developed an intracerebral hematoma from a ruptured pseudoaneurysm of the middle cerebral artery (MCA) that showed a rapid spontaneous complete thrombosis.

## Case presentation

**Figure 1:**
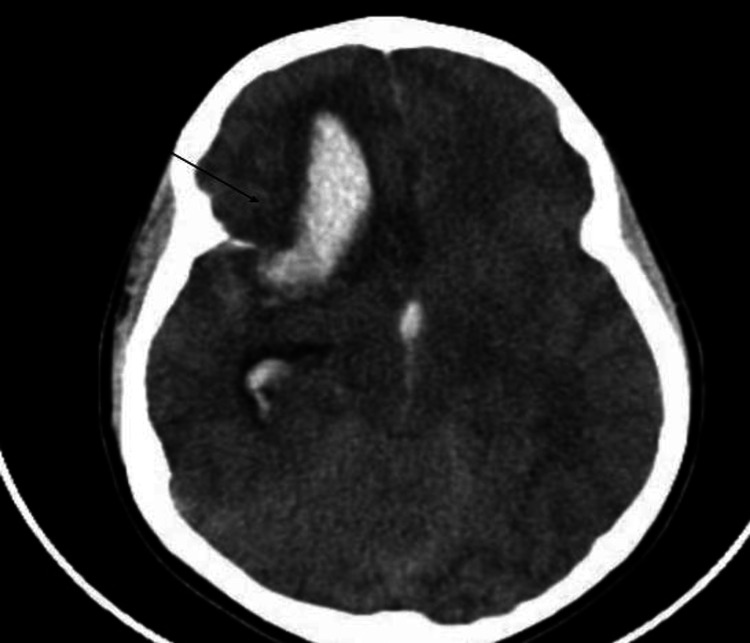
Head CT scan without contrast shows a ​​6.0 x 3.2 x 3.0 cm right frontal intracerebral hematoma (arrow) with rupture into the frontal ventricular horn extending to all ventricles

A healthy 12-year-old male with no significant past medical history or drug allergies was playing basketball at his aunt's house when he suddenly developed a severe headache accompanied by nausea and vomiting. He denied any head trauma to his aunt and mother. He was given an over-the-counter analgesic and went to bed, awakening in the early morning hours with another episode of a sudden severe headache that subsided without medication. He went back to bed, but a couple of hours later, his mother noticed that he had wet his bed. When awakened, he was drowsy and confused. His mother took him to the local hospital. He had two tonic-clonic seizure episodes immediately after his arrival and was intubated to protect his airway. A head computed tomographic (CT) scan without contrast showed a ​​6.0 x 3.2 x 3.0 cm right frontal intracerebral hematoma with rupture into the frontal ventricular horn extending into all the ventricles, causing a 1.2 cm shift of the midline (Figure 1). The patient was promptly transferred to our institution for neurosurgical evaluation. The patient arrived at our institution intubated without sedation. The physical examination demonstrated an acutely ill patient unresponsive to painful external stimuli. The head had no evidence of trauma. He did not open his eyes spontaneously or to pain. The pupils were midsize with sluggish pupillary reflexes. He had corneal and gag reflexes present.

**Figure 2:**
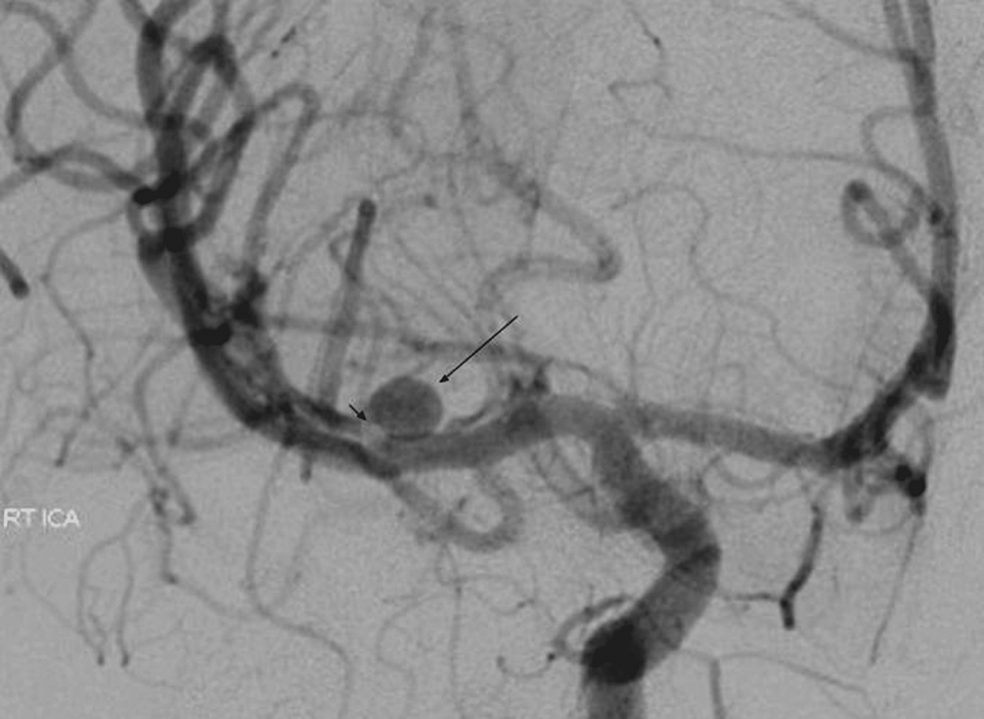
Cerebral digital subtraction angiography anteroposterior view shows a right middle cerebral artery M1 segment 7mm dissecting pseudoaneurysm (long arrow) with a filling defect in the parent artery due to the dissection of the arterial wall layers (short arrow)

**Figure 3:**
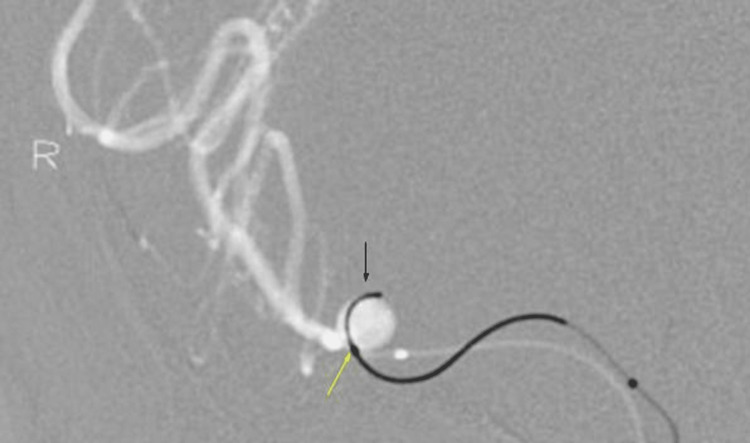
Digital subtraction angiography image in "road map" mode shows the microwire (black arrow) inside the pseudoaneurysm dome, but the microcatheter (yellow arrow) stuck in the arterial wall layers

A cerebral digital subtraction angiography (DSA) showed a dissection at the M1 segment of the right MCA with an associated 7 mm dissecting pseudoaneurysm (Figure 2). Despite multiple attempts, embolization of the pseudoaneurysm was unsuccessful because of the dissection of the artery. The microwire could be passed through the layers of the arterial wall dissection and placed inside the dome; however, the microcatheter could not be passed through the wall layers into the dome to release the coils (Figure 3). Because of the patient's critically ill condition, an attempt to clip the pseudoaneurysm was not advised as it may require the obliteration of the parent artery. An emergency left external ventricular drainage was placed, followed by a right decompressive craniotomy.

**Figure 4:**
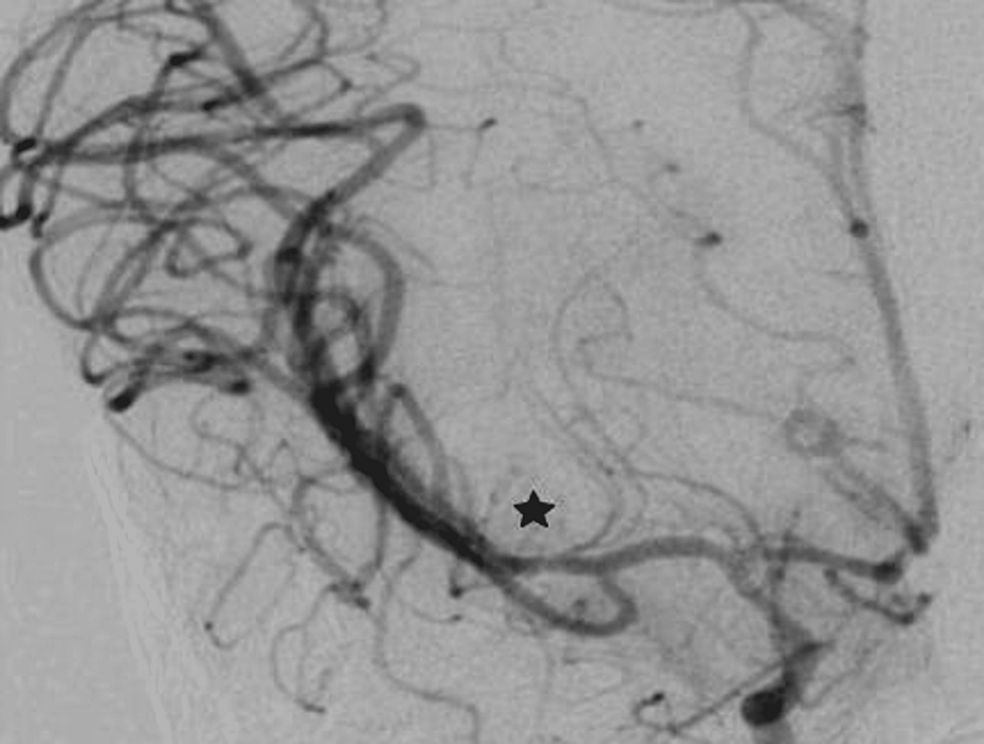
Cerebral digital subtraction angiography five days later shows no evidence of the previously observed pseudoaneurysm (a black star marks the location of the previously identified pseudoaneurysm)

**Figure 5:**
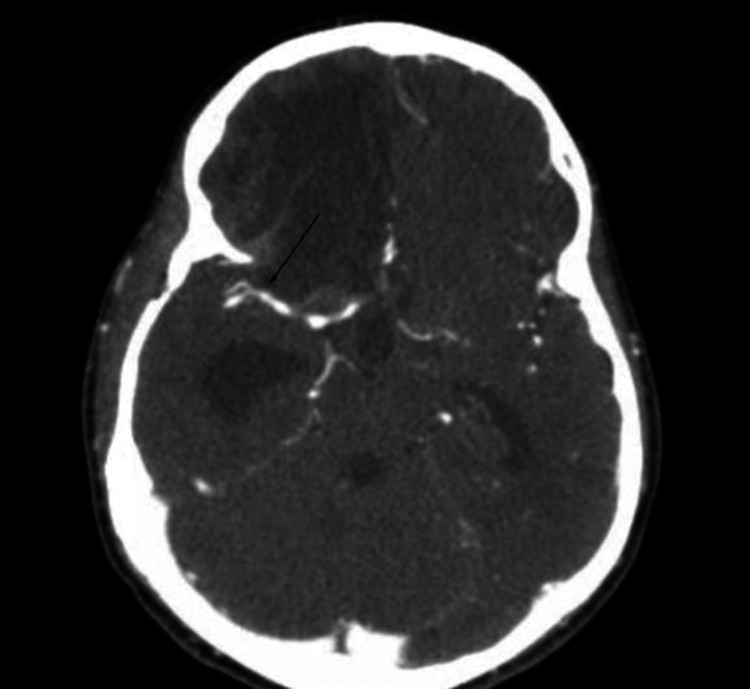
Follow-up brain CT angiography axial image performed two months after admission shows no evidence of the pseudoaneurysm (arrow indicates the location of the previously identified pseudoaneurysm) with frontal encephalomalacia and asymptomatic ex-vacuo ventriculomegaly

Five days later, the patient's condition improved, opening his eyes to the pain and making flexion withdrawal of the extremities to painful stimuli. Repeated cerebral DSA on the fifth day showed no evidence of the previously observed pseudoaneurysm (Figure 4). A tracheostomy and gastrostomy were performed 14 days after the admission, and he was weaned from the ventilator a week later. The patient neurological status slowly improved, becoming alert and oriented, communicating with facial gestures and hand movements. He had residual left hemiplegia with spasticity, which was more pronounced in the upper extremity. A follow-up CT angiography of the brain was performed two months after the admission showing no evidence of an aneurysm, and he was discharged for inpatient rehabilitation (Figure 5).

## Discussion

Most cerebral pseudoaneurysms occur after head trauma, severe infections, congenital vessel defect, hereditary connective tissue disorders, and allergic arteritis [7,10,11]. Spontaneous pseudoaneurysms are rare, and only a few cases have been described in pediatric patients [7,11-15]. Spontaneous pseudoaneurysms involving the MCA frequently produce an intracerebral hemorrhage [7,12-14]. On rare occasions, the pseudoaneurysm can be discovered before it ruptures. Yi et al. reported a 17-year-old male who complained of chronic headaches, and during the workup, an unruptured MCA spontaneous giant pseudoaneurysm was discovered [11].

The true natural history of intracranial pseudoaneurysms is unclear, and complete spontaneous thrombosis is considered an uncommon event [2,8,9,16]. The mechanism for spontaneous occlusion is unknown but may implicate parent artery remodeling or thrombus formation [2,9,16]. Songsaeng et al. showed that the most crucial factor promoting thrombosis and healing of the dissecting intracranial aneurysms was the presence of a mural hematoma which promotes healing by organizing into fibrous tissue and reducing the lumen of the dissecting aneurysm, resulting in a reduction of inflow blood, stasis of blood flow, and subsequent shrinkage of the aneurysm [9]. Anichini et al. reported on a six-year-old girl with a proximal MCA dissecting aneurysm with a spontaneous thrombosis of the aneurysm and the parent artery occurring three months later [15]. Complete or partial spontaneous thrombosis of a dissecting aneurysm occurs in 16.9% of pediatric cases and can occur up to seven months after the initial presentation [17]. Cellerini and Mangiafico reported an unusual case in which a 16-year-old female with an unruptured MCA M2-M3 segment spontaneous fusiform dissecting aneurysm showed complete thrombosis 18 months later without sequela [8].

Surgical options for pseudoaneurysms include direct clipping, suturing, wrapping-clipping, ligating the parent artery, and trapping with or without bypass [1,2,3,5,16,17]. Endovascular procedures include coiling, stent-assisted coiling, flow diversion stent, and occlusion of the parent artery [2,3,16]. Surgical and endovascular procedures carry similar in-hospital complications [3]. Patients with large hematomas require surgical drainage. Patients with intracerebral hematomas tend to rebleed, and aggressive management is typically recommended due to the considerable risk of catastrophic intracranial hemorrhage [3,17]. Conservative management can be followed for some patients with non-hemorrhagic ischemic presentations [17].

Spontaneously thrombosed pseudoaneurysms should be closely followed by neuroimaging studies to detect if partial or complete recanalization has occurred [2,9]. In children, it has been observed that parent artery occlusion can occur in up to 50% of the patients as part of the remodeling process [9]. However, cerebral ischemia is unlikely, as rich collateral circulation can be observed shortly after the parent artery occlusion has occurred [9]. For those patients managed with endovascular procedures, continued follow-up is recommended because of the possibility of recanalization [1].

## Conclusions

Spontaneous cerebral dissections or pseudoaneurysms in children are rare. Their management remains controversial, and in most cases, they are managed by surgery or endovascular techniques. In some cases, conservative management can produce thrombosis of the pseudoaneurysm. However, patients with thrombosed pseudoaneurysms must be closely followed by neuroimaging studies as they may subsequently recanalize.
